# Analysis of the temporal and spatial evolution characteristics and influencing factors of China’s herbivorous animal husbandry industry

**DOI:** 10.1371/journal.pone.0237827

**Published:** 2020-08-19

**Authors:** Chengji Han, Guogang Wang, Yongxiang Zhang, Lili Song, Lizhi Zhu

**Affiliations:** Institute of Agricultural Economics and Development, Chinese Academy of Agricultural Sciences, Beijing, China; Institute for Advanced Sustainability Studies, GERMANY

## Abstract

It is vast significance to explore the spatial and temporal evolution characteristics and influencing factors of herbivorous animal husbandry industry based on the context of China’s agriculture pursuing high-quality development. In this paper, we analyze the spatial and temporal evolution of the layout of China’s herbivorous animal husbandry industry and its influencing factors based on the spatial autocorrelation analysis, standard deviation ellipse, and spatial Durbin model with data from 1980 to 2017. The results show that there are significant positive autocorrelation characteristics of "high-high" aggregation and "low-low" aggregation in the Chinese herbivorous animal husbandry industry. To be specific, in the past four decades, the spatial distribution center of China’s herbivorous animal husbandry industry has moved towards the northeast, crossing the boundary between agriculture and animal husbandry in China, presenting a clear trend of moving from pastoral area to agricultural area; the gradual narrowing of the spatial distribution range indicates the gradually increased degree of aggregation within the industry; the east-west stretch of spatial distribution has been strengthened, and the azimuth angle shows clockwise change, suggesting that the industrial expansion in the northeast-southwest direction is prominent; the influencing factors of changes in the spatial distribution of the industry reveal that the quantity and production capacity of productive land, people’s income and living standards, and the level of mechanization will promote the development of China’s herbivorous animal husbandry industry, and are essential factors influencing industrial distribution and transfer, while policy factor has small or even not significant impact on industrial aggregation, reflecting that the policy has not played the expected role, and policy support needs to be further intensified.

## Introduction

Herbivorous animal husbandry industry is an important part of China’s modern animal husbandry and modern agriculture, and its development plays an important role in promoting the adjustment of agricultural structure, adapting to consumption upgrades and realizing the comprehensive utilization of resources. Accurately grasp the characteristics of spatial-temporal changes and the impact of causality on the development of herbivorous animal husbandry in China is a great practical significance for the preparation of scientific and effective herbivorous animal husbandry development plans and policies, thereby promote the sustainable and healthy development of herbivorous animal husbandry in China.

With the rapid development of industrialization and urbanization, and the subsequent increased population and improvements in the living standards of urban and rural residents, China’s food consumption structure for its residents has changed greatly. According to the available statistical data, the annual per capita ration consumption in China has decreased by 47% in the last 40 years (since the 1980s), while the per capita consumption of animal food, such as meat, eggs, milk, and so, has increased by 160% [1]. The long-term theories and practices have shown that the development of animal husbandry is an important and necessary measure to ensure the national food security, enrich the dietary structure of the residents, increase the incomes of farmers, and promote structural reforms in agricultural supply chains. In recent years, the *Development Plan for National Herbivorous Animal Husbandry* and the No. 1 Central Document of China have proposed to “optimize the agricultural production structure” and “develop herbivorous animal husbandry”. As a result, a foundation was laid for policies related to the subsequent development of China’s herbivorous animal husbandry. China’s available grassland areas consist of nearly 3 billion hm^2^, which is 2.5 times that of its cultivated land area. These grassland areas are mainly concentrated in the pastoral regions. Pastoral regions have always been considered to be the “main battlefields” for the development of herbivorous animal husbandry [2]. However, at the present time, the herbivorous animal husbandry industry processes in pastoral regions are facing many severe challenges, such as climate change, grassland degradation, lack of capital and technology, increased breeding costs, and so on. The industry has reached the stage of development which must accelerate industrial upgrading. In this context, explorations of the temporal and spatial evolution characteristics and influencing factors of China’s herbivorous animal husbandry are of major practical significance to the optimization of factor allocations, as well as the designs of industrial structures and spatial layouts, and the promotion of structural adjustments and mode transformations related to herbivorous animal husbandry. However, the current research studies regarding industrial agglomerations and transferences in China agricultural sectors have mainly focused on the grain fieldswhich guarantee national economic security or special economic food crops, such as cotton, vegetables, fruit, and other crops. For example, Li Z et al. [3] comprehensively used the spatial-temporal changes patterns of rice planting in China since 1949. In doing so, the analysis methods of time-series trend and spatial agglomeration were used to analysis under the influence of climate change, the results indicate the essential regular that the center of rice planting in China is moving to the northeast. Ping JL et al. [4] used cotton statistical data from 1998 to 2000 to analysis the spatial dependence of global cotton in wet and dry seasons, so as to provide a basis for exploring the regional management of cotton planting. Ji L et al. [5] used spatial Durbin model to explain the influencing factors of spatial agglomeration of Chinese vegetable industry, so as to labor force, traffic density and urban population growth had significant positive effects on vegetable production. Ren YJ et al. [6] explored the spatial agglomeration, spillover effect and green economic efficiency of the fruit industry by using the spatial Durbin model, and found that the agglomeration of the fruit industry improved the regional green economic efficiency. However, there have been few discussions to date regarding the spatial distribution characteristics and mechanisms of the spatial agglomerations and transferences in China’s herbivorous animal husbandry industry.

Industrial spatial agglomerations and distributions are considered to be the most prominent geographical features of economic activities, which are regarded as global economic geographical phenomena. The aforementioned play important roles in promoting industrial development and regional economic growth. Therefore, they have become typical topics in the fields of economic geography, regional economics, and industrial economics. The previous research methods have mainly focused on industrial concentrations, spatial Gini coefficients, gravity center models, global autocorrelations, and so on. However, the aforementioned methods are considered to be more suitable for confirmatory research projects, and can only confirm the existence of the phenomena of industrial spatial agglomerations, without revealing the characteristics of the agricultural spatial agglomerations and distributions. In actuality, decision makers have been more concerned with the locations, scopes, and trends of industrial agglomerations, along with the influencing factors of such agglomeration changes. Therefore, when compared with the confirmatory results, the exploratory spatial data analysis results have been found to be more beneficial to scientific decision-making. At the same time, it was also found in this study through research report retrieval that the existing research data regarding the impact factors of agricultural spatial agglomerations are either qualitative discussions or quantitative measurements obtained using simple classical linear regression models. For example, the influences of spatial interactions between different research units were not considered, which essentially reduced the reliability of the research results.

Therefore, based on this study’s grasp of the overall development situation of China’s herbivorous animal husbandry industry, the spatial agglomeration characteristics, spatial distribution characteristics, and evolution process of China’s herbivorous animal husbandry for the period ranging from 1980 to 2017 were systematically analyzed using spatial autocorrelation analysis and standard deviational ellipse methods. On that basis, a spatial Durbin model was used to explore the influencing factors of the spatial transferences of herbivorous animal husbandry based on the panel data. The goal of this study was to build a reasonable spatial layout and optimize the factor allocations in order to provide a scientific basis for the formulation of China’s herbivorous animal husbandry industry policies and sustainable development processes.

## Materials and methods

### Methods

#### Spatial correlation

Spatial correlation is the “first law of geography”, which means that everything is spatially correlated, and the closer the things, the greater the spatial correlation. Comprehensive spatial autocorrelation analysis is divided into two types: global measurement (Global Moran’s I) and local measurement (Anselin Local Moran’s I), which are used to determine the closeness of industrial spatial correlation and the hot and cold spots areas in space respectively. The calculation of global spatial autocorrelation and local spatial autocorrelation is shown in Eqs (1) and (2) respectively:

$$Global\;Moran\prime s\;I=\frac{n\sum_{i=1}^{n}\sum_{j=1}^{n}w_{ij}(x_{i}-\bar{x})(x_{j}-\bar{x})}{\sum_{i=1}^{n}\sum_{j=1}^{n}w_{ij}\sum_{i=1}^{n}{\left(x_{i}-\bar{x}\right)}^{2}}$$ (1)

$$Anselin\;Local\;Moran\prime s\;I=\frac{\left(x_{i}-\bar{x}\right)}{S^{2}}\underset{j}{\sum }w_{ij}(x_{j}-\bar{x})$$ (2)

Where *x*_*i*_ and *x*_*j*_ represent the production of herbivorous animal husbandry industry in the *i*-th and *j*-th spatial units; *n* is the number of selected regional units; $\overset{-}{x}$ is the mean of the production of herbivorous animal husbandry industry; *w*_*ij*_ denotes the spatial weights matrix that is expressed by Queen adjacency matrix in this study; *S* is the standard deviation of the production of herbivorous animal husbandry industry in various places. The value of *Global Moran’s I* is between [−1, 1]. When *Global Moran’s I* > 0, it represents that there is a positive correlation between the production of herbivorous animal husbandry industry in area *i* and that in neighboring area, with an aggregated distribution in space; when *Global Moran’s I* < 0, it indicates that there is a negative correlation between the production of herbivorous animal husbandry industry in area *i* and that in the neighboring area, with a scattered distribution in space; if *Global Moran’s I* = 0, it denotes that the production of herbivorous animal husbandry industry in area *i* has no correlation with that in the neighboring area, that is, there is no spatial correlation. The value of the local spatial autocorrelation *Anselin Local Moran’s I* is not limited to [−1, 1]. Positive *Anselin Local Moran’s I* means that a high value *x*_*i*_ is surrounded by its neighboring high values, which is a high-high aggregation; or it means a low value xi is surrounded by the adjacent low values, which is a low-low aggregation. Negative *Anselin Local Moran’s I* indicates that a low value *x*_*i*_ is surrounded by its neighboring high values, which belongs to low-high aggregation; or a high value *x*_*i*_ is surrounded by its adjacent low values, which is a high-low aggregation [7]. The local autocorrelation can be expressed by LISA diagram.

#### Standard deviational ellipse

The standard deviation ellipse, a method that can accurately reveal the spatial distribution features of geographical elements in spatial statistical methods, has been extensively applied in a variety of fields such as economics [8], sociology [9], and geology [10]. It quantitatively describes the spatial distribution features and evolution of industry through four parameters: coordinates, azimuth, major axis and minor axis of distribution center, among which the coordinates of the distribution center reflect the relative position of the industrial spatial distribution, the azimuth reflects the main trend direction of the industrial spatial distribution, and the comparison between the major axis and minor axis reflects the pattern of the industrial spatial distribution. This study adopts the standard deviation ellipse to reveal the spatial distribution features and evolution of China’s herbivorous animal husbandry industry.

#### Spatial durbin model

The traditional statistical theory is based on the assumption of independent observations. However, in actual research, statistical data usually not only include time dimension, but also involve space dimension. Therefore, traditional statistical methods fail to accurately identify the spatial effects when there are spatial characteristics in the data [11]. Spatial econometrics can creatively overcome the defects of classical econometric methods in processing spatial data. Compared with classical linear regression models, spatial econometrics conforms more to theory and practice, and has been widely used in many research areas. Common spatial econometrics models include spatial lag model (SAR), spatial error model (SEM), and the spatial Durbin model (SDM), among which SDM not only considers the spatial correlation between dependent variables, but also takes the spatial correlation between independent variables into account [12], making it more suitable for analyzing the spatial effects of economic things. Its basic form is:

$$\begin{array}{l} y=\rho Wy+X\beta +WX\theta +\varepsilon \\ \varepsilon \sim N(0,\sigma^{2}I_{n}) \end{array}$$ (3)

Where *y* is the dependent variable, that is, the output of herbivorous animal husbandry industry; *X* is the independent variable, that is, a series of factors affecting the development of herbivorous animal husbandry industry; *Wy* denotes the spatial lag term of the dependent variable, and *WX* is the spatial lag term of the independent variable; *ρ*, *β* and *θ* are the response parameters of dependent variable or independent variable; ε is the disturbance term vector, which is normally distributed.

### Variables and data sources

The output indicators are most commonly used for measuring the spatial dependence and spatial distribution features of an industry. The output indicators of the herbivorous animal husbandry industry generally include the breeding livestock, slaughtered, and production, among which the data of production are most frequently used, which is the most direct indicator reflecting the development of herbivorous animal husbandry industry. Considering the availability of data, furthermore beef cattle, mutton sheep and cows are the main herbivores animal in China. Therefore, this study selects beef, lamb and milk production as the output indicators.

In China, beef cattle, sheep and cows are the main herbivores. Therefore, the output of beef, mutton and milk are selected as the output indicators in this study.

In the process of industrial development, nature has provided a living space and material basis for the industries; economic society has offered a market environment for the industries. Natural, economic and social factors provide the most basic conditions for industrial development [13]. However, as a part of the agricultural industry, herbivorous animal husbandry industry also has the common characteristics of agricultural development, which include technological promotion and policy guidance [14]. Therefore, this study explores the factors influencing the evolution of the spatial structure of China’s herbivorous animal husbandry industry from four aspects: natural conditions, economic society, agricultural technology and policy support. Specifically, in terms of the natural conditions, nature provides production conditions for herbivorous animal husbandry industry through the supply of production factors and the influence of natural environment [15], while the land resource is the most direct production factor supporting the production of herbivorous animal husbandry industry [16]. At the same time, the climate change and the heterogeneity of natural conditions are also directly reflected by natural disasters [17]. Therefore, based on relevant researches, the proportion of productive land, land production capacity and the proportion of agriculture suffering disasters are selected as the three indicators to characterize the natural endowment factors that influence the development of herbivorous animal husbandry industry; regarding the economic society, existing researches show that economic development provides agricultural development with a market environment, and urban residents are the main consumers of animal products [18]. Therefore, per capita GDP, urbanization rate, and Engel coefficient of urban residents are selected to characterize the impact of the external environment of the economic society on the development of herbivorous animal husbandry industry; in agricultural production, labor force is one of the three traditional production factors [19], and mechanized operation and irrigation facility construction are the optimal choices to improve the agricultural production capacity [20]. Therefore, agricultural labor productivity, mechanization level and effective irrigation rate are used to reflect the agricultural technological factors; in terms of the policy, transportation infrastructure construction is the most basic force driving agricultural development [21]. The endorsement of the government documents also provides policy support for agricultural development. Therefore, with respect to policy factors, the transportation network density and policy dummy variable are adopted to characterize the policy support for agriculture. Among them, in order to make the policy effect more obvious and the indicators more representative, comparative and scientific, we selected the two landmark policy as a dummy policy variable as “*Regional Layout Planning of Superior Agricultural Products*” (2003) and the “*Work Program for the Pilot Grass-based Livestock Husbandry Development*” (2015), the former is an important guiding document for optimizing the layout of China’s agricultural productivity, laid the foundation for the first "*The Central No*. *1 Document*", and the latter clarifies the important position of herbivorous animal husbandry industry in the adjustment of agricultural industrial structure. The variables of spatial econometrics are described in Table 1.

**Table 1:** Description of spatial econometrics variables.

Influencing factors	Selection of concrete variable and symbol	Description of variable
**Natural conditions factors**	Proportion of productive land (%) *x*_*1*_	Sum of area of cultivated land and grassland/total land area
	Land production capacity (t/hm^2^) *x*_*2*_	Total grain output/grain sown area
	Proportion of agriculture suffering disasters (%) *x*^*3*^	Agricultural disaster area/cultivated land area
**Economic and social factors**	Per capita GDP (ten thousand yuan) *x*^*4*^	Total regional GDP/total population
	Urbanization rate (%) *x*^*5*^	Urban population/total population
	Engel coefficient of urban residents (%) *x*^*6*^	Per capita food expenditure of urban residents/total per capita consumption expenditure of urban residents
**Agricultural technological factors**	Agricultural labor productivity (ten thousand yuan / person) *x*_*7*_	Total agricultural output value / agricultural labor force
	Mechanization level (kWh/hm^2^) *x*_*8*_	Total power of agricultural machinery/cultivated land area
	Effective irrigation rate (%) *x*_*9*_	Effective irrigation area/cultivated land area
**Policy support factors**	Density of transportation network (km/10^4^ km^2^) *x*_*10*_	Total mileage per 100 square kilometers of railways and highways/total land area
	Policy dummy variable (new policy) *x*_*11*_	The policy dummy variable *x*_*11*_ is set according to the relevant provinces in the "*Work Program for the Pilot Grass-based Livestock Husbandry Development*" released by the Ministry of Agriculture and Rural Affairs in 2015 (the policy support area *x*_*11*_ = 1, non-policy-lacking area *x*_*11*_ = 0)
	Policy dummy variable (old policy) *x*_*12*_	The policy dummy variable *x*_*12*_ is set according to the relevant provinces that support beef cattle, mutton sheep and cows farming in the "*Regional Layout Planning of Superior Agricultural Products*" released by the Ministry of Agriculture and Rural Affairs in 2003 (the policy support area *x*_*12*_ = 1, non-policy-lacking area *x*_*12*_ = 0)

Among the above data, the production data come from China’s statistical database of economic and social development; the data on land, urbanization, per capita consumption, mechanization level, irrigation and transportation network are derived from China Statistical Yearbook (1981–2018) and he Ifind database of Straight Flush.

Spatial correlation analysis, Standard deviational ellipse and calculation using Spatial Durbin Model were conducted using GeoDA, ArcGIS and Stata software respectively.

## Results and analysis

### Analysis of the spatial and temporal characteristics of the industrial agglomerations of China’s herbivorous animal husbandry industry

#### Autocorrelation analysis of the spatial distributions

**Fig 1:**
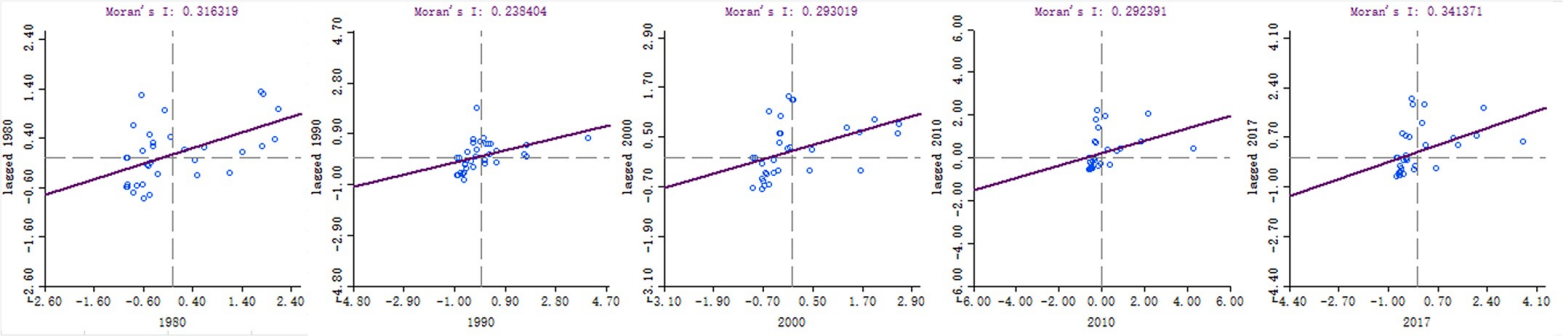
Time series changes in the global autocorrelation of herbivorous animal husbandry industry in China.

**Fig 2:**
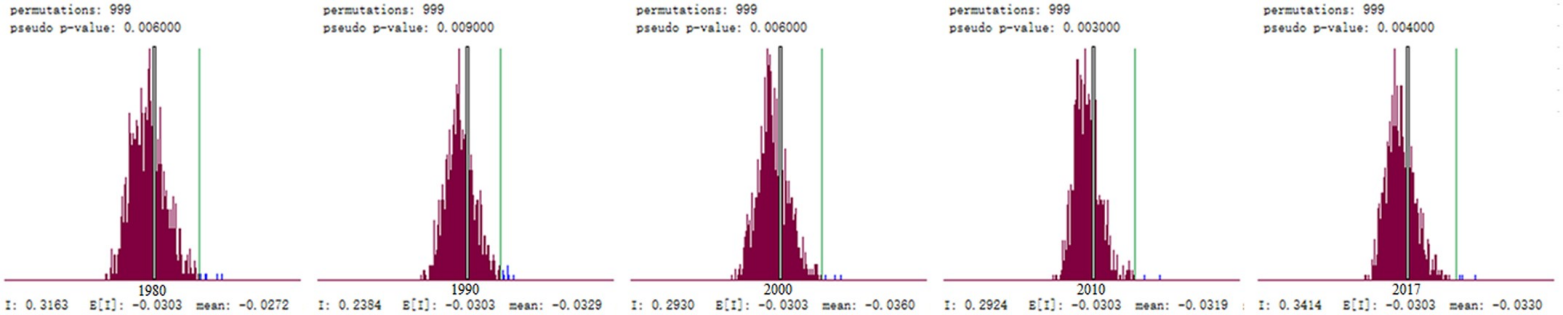
Randomized test of global autocorrelation of herbivorous animal husbandry industry in China.

As detailed in Fig 1, the years 1980, 1990, 2000, 2010, and 2017 were selected from the research period for autocorrelation analysis in this study. It was found from the examination results that the global spatial autocorrelation indices of the output of herbivorous animal products in China were all positive. Therefore, under the assumption of normal distributions, the results of index testing were also highly significant, as shown in Fig 2. This study’s findings indicated that the spatial distributions of the herbivorous animal husbandry in China were not completely random, but showed a spatial aggregation pattern among similar values. The spatial dependence features included that the provinces with higher output tended to be adjacent to the provinces with higher output. In addition, the provinces with lower output tended to be adjacent to the provinces with lower output. In other words, the neighboring provinces tended to show the "high-high" (Quadrant I) or "low-low" (Quadrant III) positive spatial autocorrelation agglomeration features. Similarly, the "low-high" (Quadrant II) or "high-low" (Quadrant IIII) displayed negative spatial autocorrelation agglomeration features, respectively.

**Fig 3:**
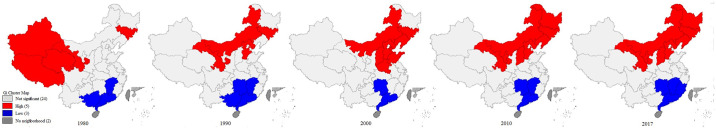
Aggregation map of Local Spatial Autocorrelation Changes (LISA) of herbivorous animal husbandry industry in China.

However, the global autocorrelation index cannot accurately reflect the spatial agglomeration characteristics of local areas. Therefore, in order to further examine the local agglomeration characteristics of China’s herbivorous animal husbandry, a LISA agglomeration map of China’s herbivorous animal husbandry was drawn at the same five time points in 1980, 1990, 2000, 2010, and 2017, respectively, as shown in Fig 3. The results showed that in 1980 the high-value agglomeration areas were mainly distributed in the Qinghai, Tibet, Xinjiang, and Gansu Provinces of western China, as well as Jilin Province in northeastern China, which was considered to be the hot spot area. Then, in 1990, Qinghai, Tibet, and Xinjiang were no longer part the high-value areas, and Inner Mongolia and Hebei Province had become the new high-value areas. Therefore, the hot spot areas displayed an obvious eastward migration trend. By the year 2000, Gansu was no longer considered to be a hot spot area. However, at that time, Shanxi, Henan, and Shandong in northern China and Liaoning in northeastern China were now part of the new hot spot areas. The hot spot areas continued to move in a northeast direction. Then, in 2010, it can be seen that the hot spot areas had continued to expand, with Gansu and Ningxia in northwestern China and Heilongjiang in northeastern China becoming the new hot sport areas. The hot spot areas in 2017 were found to be the same as those in 2010. These areas covered ten provinces and regions, specifically Gansu and Ningxia in the northwest; Hebei, Shanxi, and Inner Mongolia in the north; Heilongjiang, Jilin, and Liaoning in the northeast; Shandong in the east; and Henan in the middle region of China. However, it was observed that the low value areas displayed minimal change, and were mainly distributed in southern China. Generally speaking, in terms of the spatial distributions of China’s herbivorous animal husbandry from 1980 to 2017, the northern sections of China were the hot spot areas, and the southern sections of China were the cold spot areas. The hot spot areas experienced a trend of moving to in a northeast direction during the time sequence change.

#### Analysis of the spatial distribution characteristics

**Fig 4:**
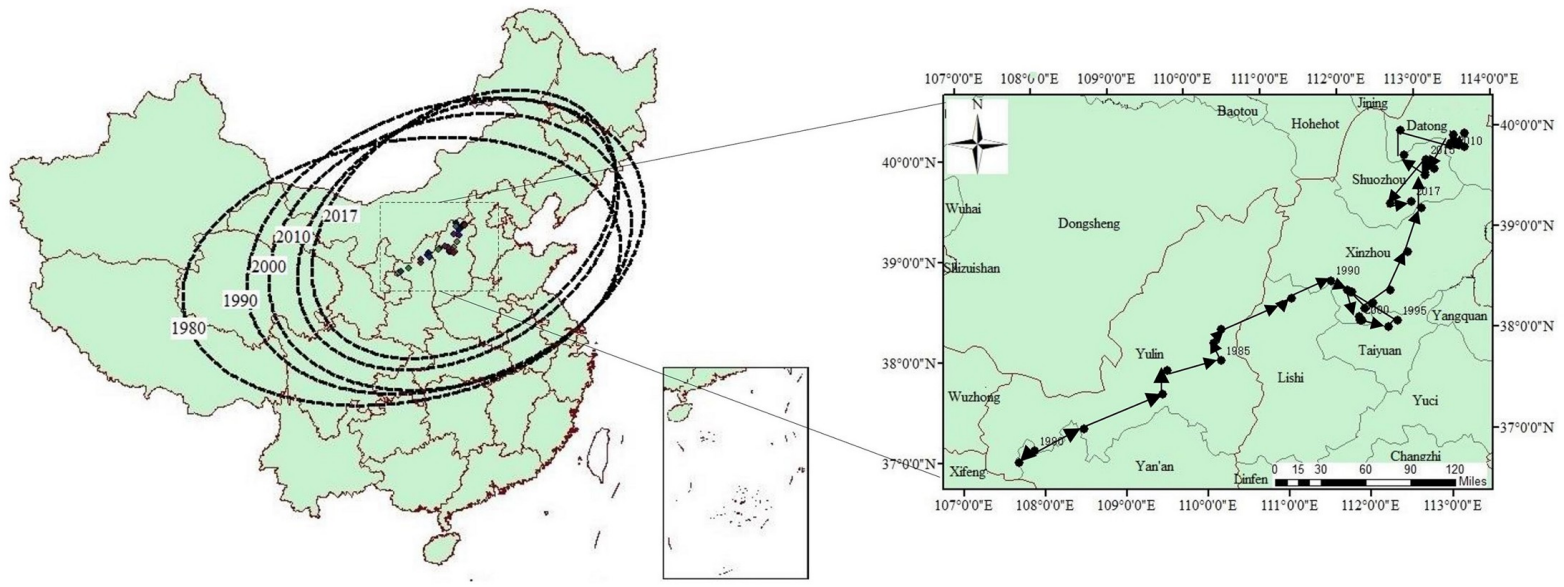
The dynamic changes in the spatial pattern of China’s herbivorous animal husbandry industry and the moving track of the center from 1980 to 2017: (a) Spatial pattern of China’s herbivorous animal husbandry industry; (b) The moving track of herbivorous animal husbandry industry center.

*Changes in the spatial distribution center locations*. Fig 4b displays the moving track of the distribution center of China’s herbivorous animal husbandry industry for the period ranging from 1980 to 2017. In can be seen in the figure that in 1980, the distribution center coordinates were (107°89′E, 37°09′N), located in Wuqi County, Yan’an City, Shaanxi Province. Then, by 2017, the distribution center coordinates were (112°85′E, 39°32′N), located in Shanyin County, Shuozhou City, Shanxi Province. Therefore, the distribution center had crossed the boundary between agricultural areas and animal husbandry areas in China. In terms of the longitude and latitude of the distribution center of China’s herbivorous animal husbandry, the change was relatively obvious along the longitude direction, and relatively small along the latitude direction. It was determined that from 1980 to 2017, the distribution center of the herbivorous animal husbandry moved by 4°96′along the longitude direction and 2°23′along the latitude direction, with a total displacement of 505.93 km. This included an eastward migration of 415.85 km and northward migration of 260.13 km. In other words, the distribution center had generally moved to the northeast. These research results were consistent with the local spatial autocorrelation changes of China’s herbivorous animal husbandry. The distribution center of China’s herbivorous animal husbandry moved in a northeast direction, which showed an obvious trend of China’s herbivorous animal husbandry transferring from the pastoral areas to the agricultural areas. As a result, China’s agricultural areas gradually became the production bases for animal products, which better realized a combination of agriculture and animal husbandry activities, and also promoted the transformation and added value of agricultural products.

*Changes of spatial distribution range*. From 1980 to 2017, the spatial distribution range of China’s herbivorous animal husbandry gradually shrank in fluctuation (Fig 4a). The standard deviation of the long axis of standard deviational ellipse decreased from 1,595.17 km in 1980 to 1,250.23 km in 2017, and the area of standard deviational ellipse decreased from 4,709,000 km2 in 1980 to 3,357,900 km2 in 2017. The spatial distribution range of China’s herbivorous animal husbandry was also reduced, thus indicating that the industrial agglomeration had gradually increased.

*Changes in the spatial distribution patterns*. From 1980 to 2017, the ratio of the short axis to the long axis of the standard deviational ellipse of the spatial distributions of the herbivorous animal husbandry industry in China showed a decreasing trend (Fig 4a), and the oblateness of the ellipse was observed to be smaller. These findings indicated that China’s herbivorous animal husbandry industry was expanding along the east-west direction and contracting along the south-north direction. In addition, the east-west expansion trend was stronger than the south-north shrinking trend.

*Changes in the spatial distribution direction*. From 1980 to 2017, the azimuth difference of the standard deviational ellipse of the spatial distributions of China’s herbivorous animal husbandry industry revealed a continuous growing trend (Fig 4a). The azimuth increased from 25.46° in 1980 to 32.21° in 2017, and the direction showed that the ellipse had rotated anticlockwise. The increase of azimuth indicated that the pull function in the northeast-southwest directions of the ellipse were enhanced, and the scale of herbivorous animal husbandry in those directions were expanding gradually.

### Analysis results of the influencing factors of spatial distributions and the evolution of the herbivorous animal husbandry industry in China

Based on the spatial dependence characteristics of the herbivorous animal husbandry industry in China, a spatial Durbin model was selected in this research study to explore the factors influencing the spatial distributions and evolution of China’s herbivorous animal husbandry. Since the Hausman test rejects the zero hypothesis that there will be no differences between the fixed effect model and the random effect model, a spatial Durbin model of fixed effects was used to analyze the influencing factors. At the same time, the spatial correlation coefficient ρ (rho) was significant and positive, and *Adj-R*^2^ = 0.987, which showed that the model had good explanatory significance. This study’s regression results are shown in Table 2.

**Table 2:** Regression results of spatial Durbin model.

Variables	Main	Direct Effect	Indirect Effect	Total Effect
x1	0.0165*** (0.0040)	0.0164*** (0.0040)	-0.0018 (0.0130)	0.0146 (0.0140)
x2	0.2010*** (0.0330)	0.2220*** (0.0270)	0.5650*** (0.0760)	0.7860*** (0.0760)
x3	0.0008 (0.0010)	0.0012 (0.0010)	0.0077*** (0.0030)	0.0089*** (0.0030)
x4	0.0893*** (0.0210)	0.0883*** (0.0230)	-0.0586 (0.0530)	0.0298 (0.0620)
x5	0.0007 (0.0010)	0.0011 (0.0020)	0.0161*** (0.005)0	0.0172*** (0.0060)
x6	-0.0136*** (0.0050)	-0.0150*** (0.0050)	-0.0408*** (0.0090)	-0.0558*** (0.0080)
x7	0.0194 (0.0330)	-0.0160 (0.0330)	-0.8750*** (0.1510)	-0.8910*** (0.1670)
x8	0.0725*** (0.0080)	0.0748*** (0.0070)	0.0713** (0.0330)	0.1460*** (0.0350)
x9	-0.0026 (0.0020)	-0.0032* (0.0020)	-0.0150** (0.0070)	-0.0182** (0.0070)
x10	-0.0001*** (0.0000)	-0.0001*** (0.0000)	0.0001 (0.0000)	-0.0001 (0.0000)
x11	0.0990 (0.0920)	0.0909 (0.0970)	-0.1580 (0.2610)	-0.0673 (0.2860)
x12	0.0221 (0.0730)	0.0233 (0.0790)	-0.0528 (0.1110)	-0.0295 (0.1190)
*ρ*	0.5060*** (0.0440)			
*Adj-R*^2^	0.9840			
Observations	1178			
Number of region	31			

***, **, and * represent that the statistics are significant at the levels of 1%, 5%, and 10% respectively.

Then, based on the fitting results of the adopted spatial Durbin model, the direct, indirect, and total effects of each influencing factor on the development of China’s herbivorous animal husbandry industry were analyzed in the current study as follows:

#### Influences of the natural conditions

The coefficient (> 0) and the direct effects of the proportions of productive land were found to be significant. However, the indirect effects were not significant, which indicated that the breadth of the lands available for the production activities of the herbivorous animal husbandry industry had directly and positively affected the production scale of the herbivorous animal husbandry industry. In addition, the grain crops, forage, and cash crops which were produced on those lands also provided sufficient agricultural and sideline product resources, such as straw for the herbivorous animal husbandry. Therefore, the coefficients, direct effects, indirect effects, and total effects of the land productivity were all considered to be positive and significant factors. However, it was found that the influences of the indirect effects were greater than those of the direct effects. These results indicated that the unit production capacities of the land were more inclined to provide favorable conditions for the herbivorous animal husbandry production activities from indirect levels. At the same time, these effects were more extensive than those of the land breadth or crop yield abundance on the development of the herbivorous animal husbandry industry. Moreover, it was found that the impacts of the agricultural disaster degrees on the herbivorous animal husbandry industry were not obvious. This study referred to the relevant research reports regarding China’s agricultural industry [22, 23], and found that natural disasters have important impacts on production output. However, for some disaster areas, the agricultural disaster degrees did not constitute significant factors for agricultural structural adjustments or the promotion of spatial transferences in the herbivorous animal husbandry industry when compared with other factors.

#### Impacts of economic and social factors

The per capita GDP and Engel coefficients of the urban residents were found to be significant in this study, among which the per capita GDP had a direct and positive impact on herbivorous animal husbandry industry. It was found that the impacts of the Engel coefficients of the urban residents on the herbivorous animal husbandry industry were observed to be negative. However, both factors reflected that the herbivorous animal husbandry industry developed with the income levels and living standards of the residents. Since the income levels and living standards of eastern China are generally higher than those of western China, this has led to a trend of China’s herbivorous animal husbandry activities transferring from the western regions to the eastern regions. When compared with the per capita GDP, it was observed that the influences of the Engel coefficients of the urban residents were more comprehensive, which indicated that the changes in living standards had exerted more prominent influences on the herbivorous animal products. In addition, when compared with the influences of the per capita GDP and Engel coefficients of the urban residents, it was determined in this study that the influences of urbanization levels on the herbivorous animal husbandry industry were not significant.

#### Influences of the agricultural technology factors

It was observed in the research results that only the effects of the mechanization levels were significant, and their direct, indirect, and total effects were all significant and positive. Meanwhile, the effects of the agricultural labor productivity and effective irrigation rates were not found to be significant. These findings suggested that only the mechanization levels of the agricultural technology play positive roles in promoting the agglomerations of herbivorous animal husbandry, and labor productivity and effective irrigation rates have limited impacting effects on the geographical agglomerations of herbivorous animal husbandry.

#### Influences of the policy support factors

Only the densities of the transportation networks were found to have significant impacts in the current research investigation. However, the impact coefficients were observed to be very small. It was found that in combination with the non-significant characteristics of policy dummy variables, the transportation infrastructure construction and policy factors had not displayed high influencing effects on the spatial agglomerations of the herbivorous animal husbandry industry. Therefore, those factors were not considered to play key roles. Previous relevant research results have shown that transportation factors do not have significant impacts on industrial agglomerations [24], which may be due to the fact that no significant differences exist in the transportation conditions throughout the Chinese provinces. Agriculture is an industry with strong sensitivity to policy issues. If the impacts of policy factors on industrial agglomerations are very small or even not significant, the most common reason will generally be that the existing policies cannot meet the industrial development requirements [25], or cannot significantly influence the production decision-making of the main agencies [26], and thereby have no significant spatial spillover effects [27]. The regression results of the policy dummy variables also show that neither the old policy nor the new policy can satisfy the industry’s development needs. Subsequently, policy factors were not considered to be important factors affecting the industrial agglomerations.

## Conclusions and discussion

In this study, spatial autocorrelation analysis and standard deviational ellipse methods were adopted in order to analyze the spatial structural changes of China’s herbivorous animal husbandry industry for the period ranging from 1980 to 2017. The key factors affecting the development and spatial structure evolution of China’s herbivorous animal husbandry industry were revealed with the assistance of a spatial Durbin Model. The results showed that there were significant positive spatial autocorrelation characteristics in China’s herbivorous animal husbandry industry. These included significant characteristics of "high-high" agglomerations and "low-low" agglomerations. A pattern of “strong in the north and weak in the south” existed for a long period of time. Then, the hot spot areas experienced a trend of moving north-east during the sequential changes. Specifically, the center of China’s herbivorous animal husbandry industry started from Wuqi County, Yan’an City, Shaanxi Province; crossed the boundaries of China’s agriculture and animal husbandry areas; and migrated toward Shanyin County, Shuozhou City, Shanxi Province. Subsequently, the elliptical coverage area became gradually reduced, indicating that the degrees of agglomerations within the industry were gradually increasing. In addition, the elliptical east-west stretching and the clockwise change of the azimuth angle indicated that the industrial scale was expanding in the east-west direction. In particular, the pulling effects from northeast to southwest continued to strengthen. The results of the various influencing factors showed that the number and production capacities of the productive land areas, along with the increased income levels and living standards of the residents and the mechanization levels, all played important roles in promoting the development of China’s herbivorous animal husbandry industry. Therefore, they were considered to be the important factors affecting the industrial layout and transferences, while the policy factors were determined to have little or no significant impacts on the industrial agglomerations. These findings reflected that policy initiatives had not played the expected roles, and the support for policy implementations requires further strengthening.

The optimization of the industrial layout of the herbivorous animal husbandry industry based on resource endowments is not only a theoretical problem which academic circles continue to discuss, but also the basis for the implementation of government policies in different areas. Based on the above-mentioned conclusions achieved in this study, the following decision-making suggestions were proposed.

First of all, the number and production capacities of productive land areas should be considered as the key factors to promote the transfer of China’s herbivorous animal husbandry activities from pastoral areas to agricultural areas. It was also revealed that the long-term overloading of grasslands, as well as serious soil degradation in pastoral areas, has forced the industry to transfer to agricultural areas with higher land production capacities. Therefore, pastoral areas should be strengthened by ecological improvements and land restoration practices in order to improve the land production capacities [28]. Meanwhile, the agricultural areas undertaking the tasks of the breeding of pastoral areas should promote combinations activities involving both planting and breeding in order to improve the stocking capacity of the land and sustainable development.

Secondly, since the income and living standards of the residents promote the development of herbivorous animal husbandry from the demand side, diversified and high-quality herbivorous animal products should be developed for different consumer groups in the future in order to meet different levels of market demand [29].

It was observed in this study that the mechanization levels promoted the development of China’s herbivorous animal husbandry industry at the agricultural technology level. However, the northern China regions were observed to be contiguous and highly mechanized, while southern China had a wide range of grassy mountain and grass slope resources. It should be considered that there should be no need for industrial hot spot areas to be limited by applicable mechanical equipment [30]. Therefore, it was recommended that mechanical production equipment should be developed which will be suitable for different regions of China in order to further improve the levels of resource utilization.

Finally, the impacts of policy factors on the herbivorous animal husbandry industry were found to be very small, and the expected policy effects had not been effectively brought into play during the examined study period. However, considering that herbivorous animal husbandry is a new type of business with significant vulnerability, the concept of raising livestock by planting grass in many regions is difficult to be reversed. The results have been low production efficiency rates in herbivorous animal husbandry production activities. Therefore, there is an urgent need to issue relevant revised policies in different regions for the purpose of building a complete policy system and promoting the sustainable and stable development of China’s herbivorous animal husbandry industry.

Based on the analysis structure of the intersections of the spatial correlation method, along with the spatial statistical method and econometric model of the economics results, this study provided a new research paradigm for the analysis of the characteristics and mechanisms of industrial agglomerations, layouts, and development patterns. In addition, this study’s results also have potential reference significance for other industry related research investigations. Although some interesting findings were achieved regarding industrial layout issues in this study, there was still considered to be space for further improvement. Subsequently, the introduced new research ideas and system improvements may potentially be proven to be completely effective only through practical testing procedures. There were also problems encountered during the analyses of the frameworks of the temporal and spatial evolution characteristics, as well as issues regarding the influencing factors of China’s herbivorous animal husbandry industry in this research. With the development of the Chinese economy and changes in the population’s food consumption structure, China’s herbivorous animal husbandry industry has received increasing attention. When compared with other basic agricultural related industries, herbivorous animal husbandry is still a new industry in China. Therefore, the acquisition of relevant statistical data, and the construction of an effective index system, present the current challenges to research endeavors. In order to address these short-comings, it is recommended that the research related to the industrial layout of China’s herbivorous animal husbandry industry be continuously enriched and improved according to the practical results, and also continuously tracked and studied.

## Supporting information

S1 Data Data of herbivorous animal husbandry of verity of provinces (1980–2017) in China. (XLSX)
